# The genome sequences of the diplonemid protist *Rhynchopus euleeides* YPF1915 and its bacterial endosymbiont *Candidatus* Syngnamydia salmonis (Chlamydiota)

**DOI:** 10.12688/wellcomeopenres.24014.1

**Published:** 2025-05-07

**Authors:** Daria Tashyreva, Drahomíra Faktorová, Aleš Horák, Julius Lukeš, John M. Archibald, Graeme Oatley, Elizabeth Sinclair, Camilla Santos, Michael Paulini, Eerik Aunin, Noah Gettle, Haoyu Niu, Victoria McKenna, Rebecca O’Brien, Wellcome Sanger Institute Tree of Life Management, Samples and Laboratory Team, Wellcome Sanger Institute Scientific Operations: Sequencing Operations, Wellcome Sanger Institute Tree of Life Core Informatics Team, EBI Aquatic Symbiosis Genomics Data Portal Team, Aquatic Symbiosis Genomics Project Leadership

**Affiliations:** 1 Institute of Parasitology, Biology Centre, České Budějovice, Czech Republic; 2 Dalhousie University Department of Biochemistry and Molecular Biology, Halifax, Nova Scotia, Canada; 3 Dalhousie Institute for Comparative Genomics, Dalhousie University, Halifax, Nova Scotia, Canada; 4 Wellcome Sanger Institute, Hinxton, England, UK

**Keywords:** Rhynchopus euleeides, Diplonemea, Euglenozoa, bacterial endosymbionts, genome sequence, chromosomal

## Abstract

We present a genome assembly of the diplonemid *Rhynchopus euleeides* (Euglenozoa; Diplonemea; Diplonemea; Diplonemidae). The genome sequence is 199.0 megabases long, with most of the assembly scaffolded into 88 chromosomal pseudomolecules. The multipartite mitochondrial genome and the 2.0 megabase genome of *Ca.* Syngnamydia salmonis, a bacterial endosymbiont of *R. euleeides*, were also sequenced and assembled.

## Species taxonomy: host

Eukaryota; Discoba; Euglenozoa; Diplonemea; Diplonemidae; *Rhynchopus; Rhynchopus euleeides* (NCBI:txid630703).

## Species taxonomy: endosymbiont

Bacteria; Chlamydiota; Chlamydiia; Parachlamydiales; Simkaniaceae; *Candidatus* Syngnamydia; *Candidatus* Syngnamydia salmonis (NCBI:txid 504270).

## Background

*Rhynchopus euleeides* is a unicellular eukaryote belonging to the class Diplonemea (commonly referred to as diplonemids) of the phylum Euglenozoa. Diplonemids are a group of heterotrophic, predominantly marine flagellates that are closely related to kinetoplastid and euglenid protists ( Kostygov *et al.*, 2021). Diplonemidae is a species-rich group, and environmental metabarcoding has revealed their high abundance in the world’s oceans ( Flegontova *et al.*, 2020). Yet despite their apparent ecological importance, diplonemids remain one of the most understudied groups of protists; fewer than two dozen species have been brought into culture ( Tashyreva *et al.*, 2022), and, prior to the current project, only one draft diplonemid genome sequence was available ( Valach *et al.*, 2023).

*R. euleeides* YPF1915 was isolated in 2019 from a seawater aquarium at the Keikyu-Aburatsubo Marine Park in Kanagawa, Japan. This relatively small protist (~25 µm long) has a cylindrical body with a slightly constricted anterior end and a rounded posterior end. Similar to other diplonemids, *R. euleeides* cells are covered by a naked plasma membrane and are capable of gliding motility and metabolic deformations that result in drastic changes of cell shape. *R. euleeides* is characterised by two distinct life stages. Under nutrient-rich conditions (seawater supplemented with blood serum), it exists in a so-called trophic stage with larger cells, prominent lipid inclusions and short flagellar stubs lacking conventional axonemes that are fully concealed inside the flagellar pocket. A smaller, fast-swimming stage with long conventional flagella develops during nutrient starvation.

During a routine screening of new diplonemid isolates for the presence of endosymbionts, we found abundant intracellular bacteria inside *R. euleeides* that were identified as *Candidatus Syngnamydia salmonis*. Diplonemids frequently form associations with alphaproteobacteria, namely members of Holosporaceae and Rickettsiaceae ( George *et al.*, 2020), but this is the first documented case of a chlamydial symbiont among diplonemids. Interestingly, this species (>98.5% similarity to *Ca.* Syngnamydia salmonis by its 18S rRNA gene) can infect a wide range of hosts, including fish, amoebozoans, and diplonemids. Sequencing of the host and the symbiont genome will provide invaluable information about diplonemid metabolism, sheds the light on the reasons for their ecological success, and helps to identify the role of chlamydial endosymbionts and molecular mechanisms of its interaction with the host.

## Genome sequence report

**Figure 1.**
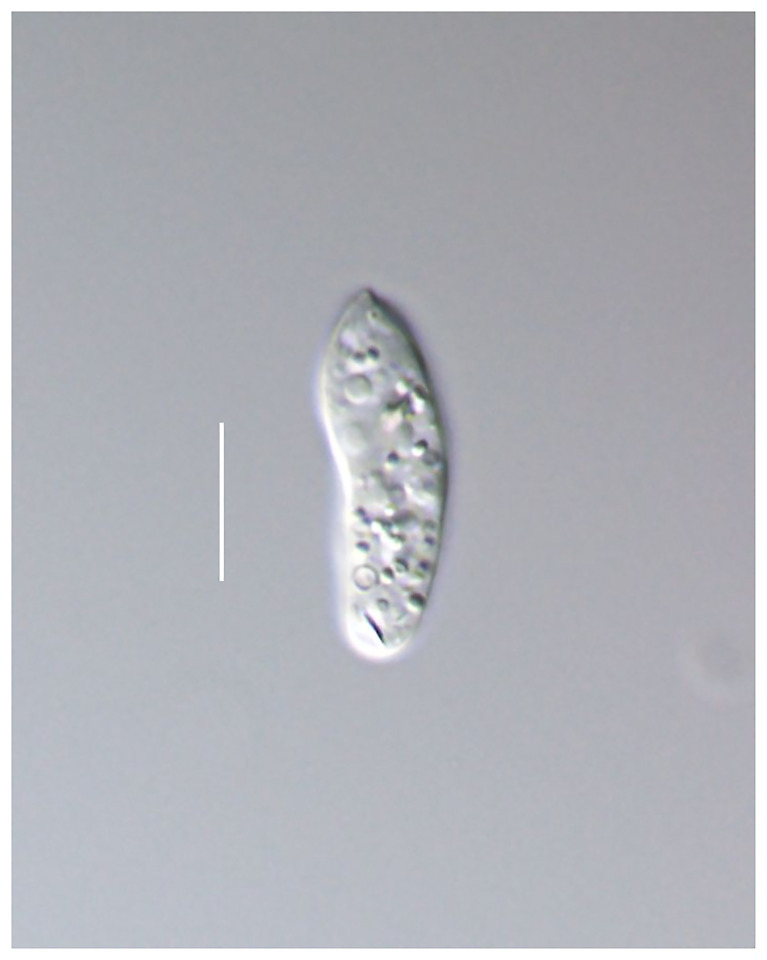
Photograph of *Rhynchopus euleeides* (peRhyEule1). Scale bar = 10 µm.

The genome sequences were generated from a sample of *Rhynchopus euleeides* YPF1915 ( Figure 1) cultured at the Biology Centre, Czech Republic. A total of 76-fold coverage in Pacific Biosciences single-molecule HiFi long was generated. Primary assembly contigs were scaffolded with chromosome conformation Hi-C data. Manual assembly curation corrected 232 missing joins or mis-joins and removed 8 haplotypic duplications, increasing the scaffold number by 45.10%, and the scaffold N50 by 0.35%.

**Figure 2.**
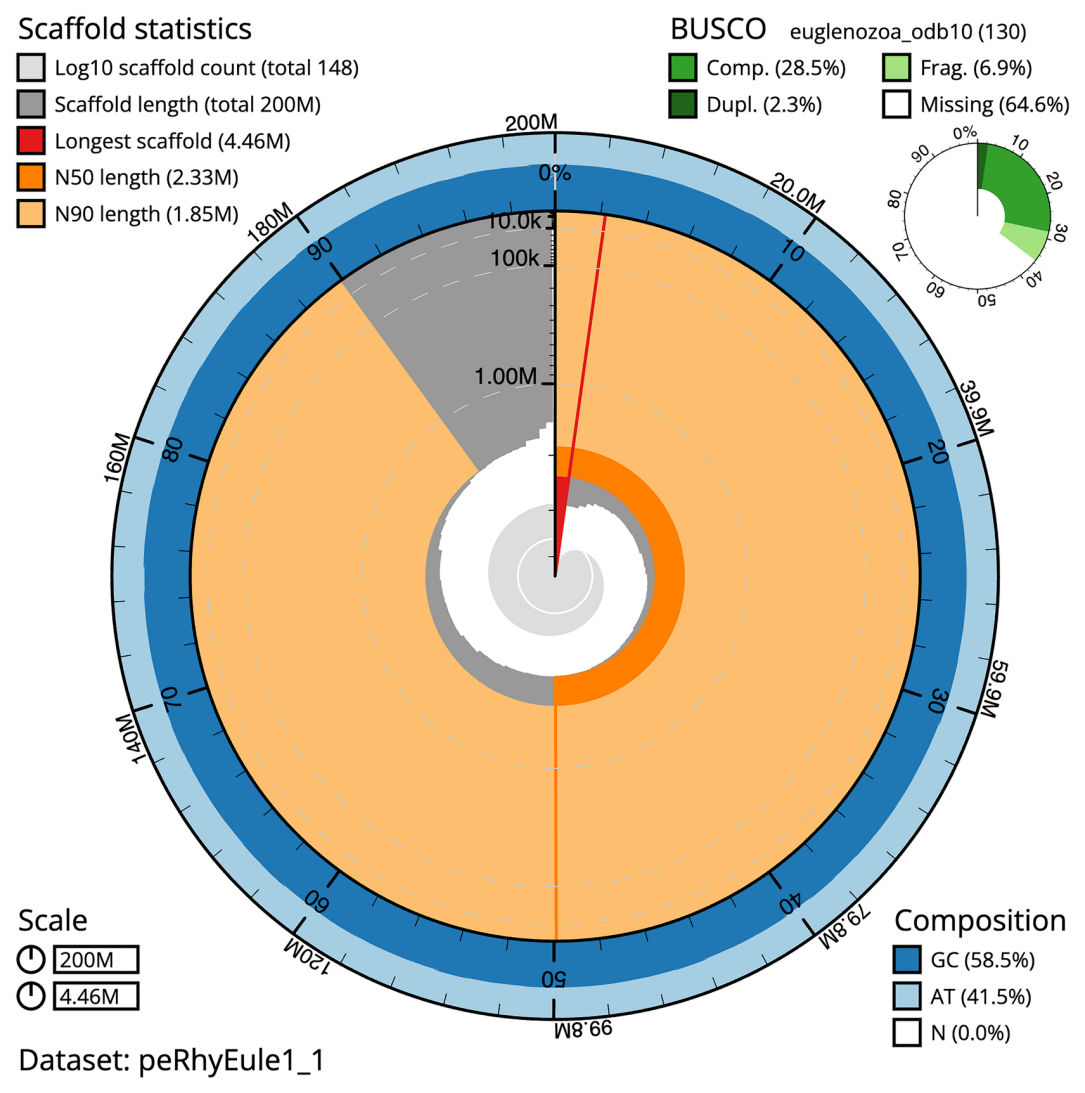
Genome assembly of *Rhynchopus euleeides*, peRhyEule1.1: metrics. The BlobToolKit snail plot shows N50 metrics and BUSCO gene completeness. The main plot is divided into 1,000 size-ordered bins around the circumference with each bin representing 0.1% of the 199,566,941 bp assembly. The distribution of scaffold lengths is shown in dark grey with the plot radius scaled to the longest scaffold present in the assembly (4,463,607 bp, shown in red). Orange and pale-orange arcs show the N50 and N90 scaffold lengths (2,330,496 and 1,853,943 bp), respectively. The pale grey spiral shows the cumulative scaffold count on a log scale with white scale lines showing successive orders of magnitude. The blue and pale-blue area around the outside of the plot shows the distribution of GC, AT and N percentages in the same bins as the inner plot. A summary of complete, fragmented, duplicated and missing BUSCO genes in the euglenozoa_odb10 set is shown in the top right. An interactive version of this figure is available at https://blobtoolkit.genomehubs.org/view/peRhyEule1_1/dataset/peRhyEule1_1/snail.

**Figure 3.**
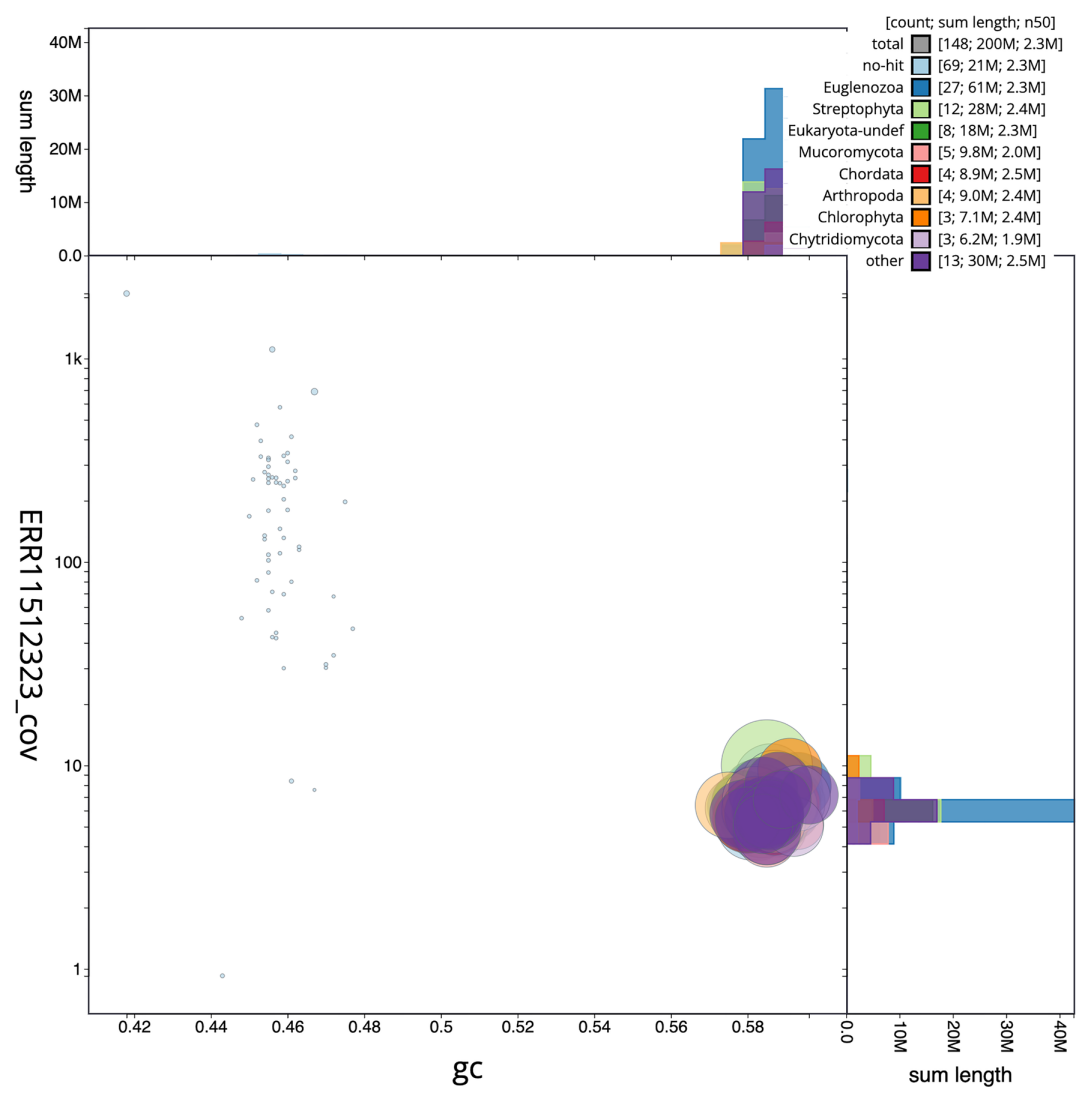
Genome assembly of *Rhynchopus euleeides*, peRhyEule1.1: BlobToolKit GC-coverage plot. Scaffolds are coloured by phylum. Circles are sized in proportion to scaffold length. Histograms show the distribution of scaffold length sum along each axis. An interactive version of this figure is available at https://blobtoolkit.genomehubs.org/view/peRhyEule1_1/dataset/peRhyEule1_1/blob.

**Figure 4.**
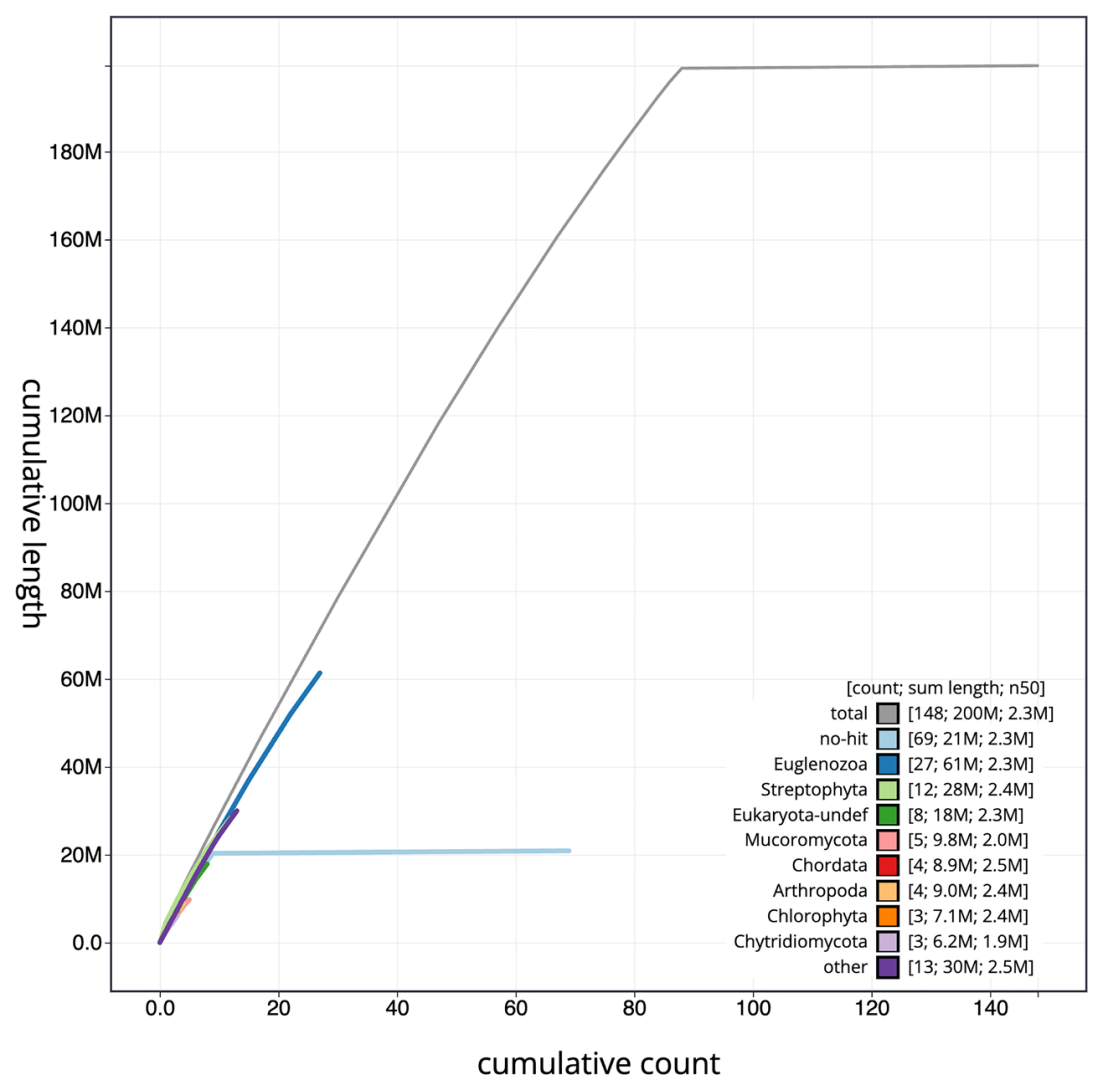
Genome assembly of *Rhynchopus euleeides*, peRhyEule1.1: BlobToolKit cumulative sequence plot. The grey line shows cumulative length for all scaffolds. Coloured lines show cumulative lengths of scaffolds assigned to each phylum using the buscogenes taxrule. An interactive version of this figure is available at https://blobtoolkit.genomehubs.org/view/peRhyEule1_1/dataset/peRhyEule1_1/cumulative.

**Figure 5.**
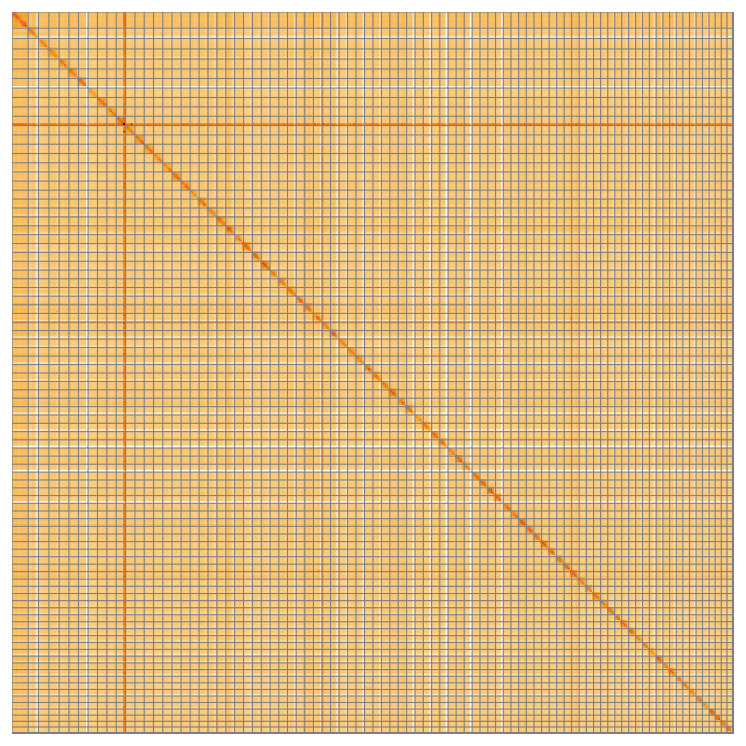
Genome assembly of *Rhynchopus euleeides*, peRhyEule1.1: Hi-C contact map of the peRhyEule1.1 assembly, visualised using HiGlass. Chromosomes are shown in order of size from left to right and top to bottom. An interactive version of this figure may be viewed at https://genome-note-higlass.tol.sanger.ac.uk/l/?d=KG1iOPTURcel7bsUk_PQ0A.

The final assembly has a total length of 199.0 Mb in 88 sequence scaffolds with a scaffold N50 of 2.3 Mb ( Table 1). The snail plot in Figure 2 provides a summary of the assembly statistics, while the distribution of assembly scaffolds on GC proportion and coverage is shown in Figure 3. The cumulative assembly plot in Figure 4 shows curves for subsets of scaffolds assigned to different phyla. Most (99.7%) of the assembly sequence was assigned to 88 chromosomal-level scaffolds. Chromosome-scale scaffolds confirmed by the Hi-C data are named in order of size ( Figure 5; Table 2). While not fully phased, the assembly deposited is of one haplotype. Contigs corresponding to the second haplotype have also been deposited. Mitochondrial scaffolds were also assembled and can be found as contigs within the multifasta file of the genome submission. The mitogenome in this species is multipartite, composed of a large number of circular fragments containing parts of each mitochondrial gene ( Valach *et al.*, 2017).

**Table 1.** Genome data for *Rhynchopus euleeides*, peRhyEule1.1.

Project accession data			
Assembly identifier	peRhyEule1.1	
Species	*Rhynchopus euleeides*	
Specimen	peRhyEule1	
NCBI taxonomy ID	630703	
BioProject	PRJEB62740	
BioSample ID	SAMEA13602747	
Isolate information	peRhyEule1 cell pellet (DNA, Hi-C and RNA sequencing)	
Assembly metrics *			
Consensus quality (QV)	59.9	
*k*-mer completeness	99.99%	
BUSCO **	C:28.5%[S:26.2%,D:2.3%], F:6.9%,M:64.6%,n:130	
Percentage of assembly mapped to chromosomes	99.7%	
Organelles	Mitochondrial genome: multipartite	
Raw data accessions			
PacificBiosciences SEQUEL II	ERR11512323, ERR11512324	
Hi-C Illumina	ERR11526213, ERR11526214	
PolyA RNA-Seq Illumina	ERR12708748	
Genome assembly			
Assembly accession	GCA_963210315.1	
*Accession of alternate haplotype*	GCA_963210325.1	
Span (Mb)	199.0	
Number of contigs	525	
Contig N50 length (Mb)	0.6	
Number of scaffolds	88	
Scaffold N50 length (Mb)	2.3	
Longest scaffold (Mb)	4.46	

* Assembly metric benchmarks are adapted from column VGP-2020 of “Table 1: Proposed standards and metrics for defining genome assembly quality” from ( Rhie *et al.*, 2021). ** BUSCO scores based on the euglenozoa_odb10 BUSCO set using version 5.3.2. C = complete [S = single copy, D = duplicated], F = fragmented, M = missing, n = number of orthologues in comparison. A full set of BUSCO scores is available at https://blobtoolkit.genomehubs.org/view/peRhyEule1_1/dataset/peRhyEule1_1/busco.

**Table 2.** Chromosomal pseudomolecules in the genome assembly of *Rhynchopus euleeides*, peRhyEule1.

INSDC accession	Chromosome	Length (Mb)	GC%
OY723237.1	1	4.46	58.5
OY723238.1	2	2.87	58.5
OY723239.1	3	2.87	58.5
OY723240.1	4	2.8	58.5
OY723241.1	5	2.71	58.0
OY723242.1	6	2.65	59.0
OY723243.1	7	2.63	58.5
OY723244.1	8	2.62	58.5
OY723245.1	9	2.59	58.5
OY723246.1	10	2.58	58.5
OY723247.1	11	2.58	58.5
OY723248.1	12	2.58	58.0
OY723249.1	13	2.56	58.5
OY723250.1	14	2.56	58.5
OY723251.1	15	2.56	58.0
OY723252.1	16	2.53	58.0
OY723253.1	17	2.52	58.5
OY723254.1	18	2.51	58.5
OY723255.1	19	2.48	59.0
OY723256.1	20	2.47	58.5
OY723257.1	21	2.46	58.5
OY723258.1	22	2.45	59.0
OY723259.1	23	2.45	59.5
OY723260.1	24	2.45	58.0
OY723261.1	25	2.45	58.0
OY723262.1	26	2.44	58.5
OY723263.1	27	2.42	58.5
OY723264.1	28	2.42	58.5
OY723265.1	29	2.41	58.0
OY723266.1	30	2.39	58.5
OY723267.1	31	2.38	57.5
OY723268.1	32	2.38	58.5
OY723269.1	33	2.37	58.5
OY723270.1	34	2.36	58.0
OY723271.1	35	2.35	59.5
OY723272.1	36	2.35	58.5
OY723273.1	37	2.35	58.5
OY723274.1	38	2.34	58.5
OY723275.1	39	2.33	58.5
OY723276.1	40	2.33	58.5
OY723277.1	41	2.33	58.0
OY723278.1	42	2.32	58.5
OY723279.1	43	2.32	59.0
OY723280.1	44	2.31	58.0
OY723281.1	45	2.3	58.0
OY723282.1	46	2.3	58.5
OY723283.1	47	2.26	59.0
OY723284.1	48	2.24	58.5
OY723285.1	49	2.24	58.0
OY723286.1	50	2.23	59.0
OY723287.1	51	2.18	59.0
OY723288.1	52	2.18	58.0
OY723289.1	53	2.17	58.5
OY723290.1	54	2.14	58.5
OY723291.1	55	2.13	58.5
OY723292.1	56	2.13	59.0
OY723293.1	57	2.12	58.5
OY723294.1	58	2.12	59.0
OY723295.1	59	2.11	59.0
OY723296.1	60	2.1	59.0
OY723297.1	61	2.09	58.5
OY723298.1	62	2.07	57.5
OY723299.1	63	2.07	59.0
OY723300.1	64	2.07	59.0
OY723301.1	65	2.06	59.0
OY723302.1	66	2.0	58.5
OY723303.1	67	1.99	58.5
OY723304.1	68	1.96	58.0
OY723305.1	69	1.96	58.5
OY723306.1	70	1.93	59.0
OY723307.1	71	1.92	58.5
OY723308.1	72	1.92	58.5
OY723309.1	73	1.92	59.0
OY723310.1	74	1.91	58.5
OY723311.1	75	1.91	58.5
OY723312.1	76	1.87	58.5
OY723313.1	77	1.85	59.5
OY723314.1	78	1.85	58.5
OY723315.1	79	1.84	58.5
OY723316.1	80	1.82	58.5
OY723317.1	81	1.82	59.0
OY723318.1	82	1.81	58.5
OY723319.1	83	1.8	58.5
OY723320.1	84	1.79	57.5
OY723321.1	85	1.71	59.0
OY723322.1	86	1.7	58.0
OY723323.1	87	1.57	58.0
OY723324.1	88	1.48	59.5
OY723325.1	MT1	0.01	45.5
OY723326.1	MT2	0.01	46.0
OY723327.1	MT3	0.01	45.5
OY723328.1	MT4	0.01	46.0
OY723329.1	MT5	0.01	46.0
OY723330.1	MT6	0.01	46.0
OY723331.1	MT7	0.01	45.5
OY723332.1	MT8	0.01	46.0
OY723333.1	MT9	0.01	45.5
OY723334.1	MT10	0.01	46.0
OY723335.1	MT11	0.01	46.0
OY723336.1	MT12	0.01	46.0
OY723337.1	MT13	0.01	46.0
OY723338.1	MT14	0.01	45.5
OY723339.1	MT15	0.01	45.5
OY723340.1	MT16	0.01	46.0
OY723341.1	MT17	0.01	45.5
OY723342.1	MT18	0.01	45.5
OY723343.1	MT19	0.01	46.0
OY723344.1	MT20	0.03	46.5
OY723345.1	MT21	0.01	46.0
OY723346.1	MT22	0.01	46.0
OY723347.1	MT23	0.01	46.0
OY723348.1	MT24	0.01	45.5
OY723349.1	MT25	0.01	45.0
OY723350.1	MT26	0.01	46.0
OY723351.1	MT27	0.01	45.0
OY723352.1	MT28	0.01	47.0
OY723353.1	MT29	0.01	45.5
OY723354.1	MT30	0.01	45.5
OY723355.1	MT31	0.01	45.5
OY723356.1	MT32	0.01	45.5
OY723357.1	MT33	0.01	45.5
OY723358.1	MT34	0.02	42.0
OY723359.1	MT35	0.01	46.5
OY723360.1	MT36	0.01	46.0
OY723361.1	MT37	0.01	45.5
OY723362.1	MT38	0.01	46.0
OY723363.1	MT39	0.01	46.0
OY723364.1	MT40	0.01	46.0
OY723365.1	MT41	0.01	45.5
OY723366.1	MT42	0.01	45.5
OY723367.1	MT43	0.01	45.5
OY723368.1	MT44	0.01	45.0
OY723369.1	MT45	0.01	47.0
OY723370.1	MT46	0.01	47.5
OY723371.1	MT47	0.01	45.5
OY723372.1	MT48	0.01	45.5
OY723373.1	MT49	0.01	46.0
OY723374.1	MT50	0.01	45.5
OY723375.1	MT51	0.02	46.0
OY723376.1	MT52	0.01	47.0
OY723377.1	MT53	0.01	46.0
OY723378.1	MT54	0.01	45.5
OY723379.1	MT55	0.01	44.5
OY723380.1	MT56	0.01	46.5
OY723381.1	MT57	0.01	46.0
OY723382.1	MT58	0.01	47.0
OY723383.1	MT59	0.01	47.0
OY723384.1	MT60	0.01	45.5

The estimated Quality Value (QV) of the final assembly is 59.9 with *k*-mer completeness of 99.99%. Using the euglenozoa_odb10 reference set ( *n* = 130), the assembly has a BUSCO v5.3.2 completeness of 28.5% (single = 26.2%, duplicated = 2.3%). The *k-*mer completeness score suggests that the assembly covers the genome almost completely with few missing sequences. The low BUSCO coverage may be attributed to factors such as a poor orthologue lineage set for Euglenozoa, with inadequate representation of diplonemids. Additionally, the gene-finding algorithms in BUSCO may not identify gene features accurately in diplonemids.

The genome of a bacterial cobiont, *Candidatus* Syngnamydia salmonis (Chlamydiota), was assembled in four scaffolds, spanning 2 Mb (GCA_963457595.1). This genome could not be circularised.

## Methods

### Sample acquisition

*Rhynchopus euleeides* YPF1915 cells (specimen ID DU0000013, ToLID peRhyEule1) were grown axenically in seawater-based Hemi medium supplemented with 1% v/v horse blood serum at the Institute of Parasitology, Biology Centre (České Budějovice, Czech Republic). Cells were collected by centrifugations on 2021-05-12 by centrifugation. The sample was collected and identified by Daria Tashyreva (Institute of Parasitology, Biology Centre) and preserved by snap-freezing the pellet.

### Nucleic acid extraction

The workflow for high molecular weight (HMW) DNA extraction at the Wellcome Sanger Institute (WSI) Tree of Life Core Laboratory includes a sequence of core procedures: sample preparation, sample homogenisation, DNA extraction, fragmentation, and clean-up. In sample preparation, the peRhyEule1 sample was weighed and dissected on dry ice ( Jay *et al.*, 2023). The cell pellet was homogenised using a PowerMasher II tissue disruptor ( Denton *et al.*, 2023a). HMW DNA was extracted using the Manual MagAttract v1 protocol ( Strickland *et al.*, 2023b). DNA was sheared into an average fragment size of 12–20 kb in a Megaruptor 3 system ( Todorovic *et al.*, 2023). Sheared DNA was purified by solid-phase reversible immobilisation ( Strickland *et al.*, 2023a), using AMPure PB beads to eliminate shorter fragments and concentrate the DNA. The concentration of the sheared and purified DNA was assessed using a Nanodrop spectrophotometer and Qubit Fluorometer and Qubit dsDNA High Sensitivity Assay kit. Fragment size distribution was evaluated by running the sample on the FemtoPulse system.

RNA was extracted from cells of peRhyEule1 in the Tree of Life Laboratory at the WSI using the RNA Extraction: Automated MagMax™ *mir*Vana protocol ( do Amaral *et al.*, 2023). The RNA concentration was assessed using a Nanodrop spectrophotometer and a Qubit Fluorometer using the Qubit RNA Broad-Range Assay kit. Analysis of the integrity of the RNA was done using the Agilent RNA 6000 Pico Kit and Eukaryotic Total RNA assay.

Protocols developed by the WSI Tree of Life laboratory are publicly available on protocols.io ( Denton *et al.*, 2023b).

### Sequencing

Pacific Biosciences HiFi circular consensus DNA sequencing libraries were constructed according to the manufacturers’ instructions. Poly(A) RNA-Seq libraries were constructed using the NEB Ultra II RNA Library Prep kit. DNA and RNA sequencing was performed by the Scientific Operations core at the WSI on Pacific Biosciences SEQUEL II (HiFi) and Illumina NovaSeq 6000 (RNA-Seq) instruments. Hi-C data were also generated from cells of peRhyEule1 using the Arima2 kit and sequenced on the Illumina NovaSeq 6000, Illumina NovaSeq 6000 instrument.

### Genome assembly, curation and evaluation

Assembly was carried out with Hifiasm ( Cheng *et al.*, 2021) and haplotypic duplication was identified and removed with purge_dups ( Guan *et al.*, 2020). The assembly was then scaffolded with Hi-C data ( Rao *et al.*, 2014) using YaHS ( Zhou *et al.*, 2023). Manual curation was performed using JBrowse2 ( Diesh *et al.*, 2023), HiGlass ( Kerpedjiev *et al.*, 2018) and Pretext ( Harry, 2022). The mitochondrial genome was assembled using MitoHiFi ( Uliano-Silva *et al.*, 2023), which runs MitoFinder ( Allio *et al.*, 2020) and uses these annotations to select the final mitochondrial contig and to ensure the general quality of the sequence.

The bacterial cobiont assembly was generated using metaMDBG ( Benoit *et al.*, 2024), binned using MetaBAT2 ( Kang *et al.*, 2019) and optimised using DAS Tool ( Sieber *et al.*, 2018). PROKKA ( Seemann, 2014) was used to identify tRNAs and rRNAs, CheckM (checkM_DB release 2015-01-16) was used to assess bin completeness/contamination, and GTDB-TK (GTDB release 214) ( Chaumeil *et al.*, 2022) was used for taxonomic classification.

### Assembly quality assessment

The Merqury.FK tool ( Rhie *et al.*, 2020), run in a Singularity container ( Kurtzer *et al.*, 2017), was used to evaluate assembly quality {for the primary and alternate haplotypes using *k*-mer databases ( *k* = 31) computed prior to genome assembly. The analysis outputs included assembly QV and *k*-mer completeness.

A Hi-C contact map was produced for the final version of the assembly. The Hi-C reads were aligned using bwa-mem2 ( Vasimuddin *et al.*, 2019) and the alignment files were combined using SAMtools ( Danecek *et al.*, 2021). The Hi-C alignments were converted into a contact map using BEDTools ( Quinlan & Hall, 2010) and the Cooler tool suite ( Abdennur & Mirny, 2020). The contact map was visualised in HiGlass ( Kerpedjiev *et al.*, 2018).

The blobtoolkit pipeline is a Nextflow ( Di Tommaso *et al.*, 2017) port of the previous Snakemake Blobtoolkit pipeline ( Challis *et al.*, 2020). It aligns the PacBio reads in SAMtools and minimap2 ( Li, 2018) and generates coverage tracks for regions of fixed size. In parallel, it queries the GoaT database ( Challis *et al.*, 2023) to identify all matching BUSCO lineages to run BUSCO ( Manni *et al.*, 2021). For the three domain-level BUSCO lineages, the pipeline aligns the BUSCO genes to the UniProt Reference Proteomes database ( Bateman *et al.*, 2023) with DIAMOND blastp ( Buchfink *et al.*, 2021). The genome is also divided into chunks according to the density of the BUSCO genes from the closest taxonomic lineage, and each chunk is aligned to the UniProt Reference Proteomes database using DIAMOND blastx. Genome sequences without a hit are chunked using seqtk and aligned to the NT database with blastn ( Altschul *et al.*, 1990). The blobtools suite combines all these outputs into a blobdir for visualisation.

The blobtoolkit pipeline was developed using nf-core tooling ( Ewels *et al.*, 2020) and MultiQC ( Ewels *et al.*, 2016), relying on the Conda package manager, the Bioconda initiative ( Grüning *et al.*, 2018), the Biocontainers infrastructure ( da Veiga Leprevost *et al.*, 2017), as well as the Docker ( Merkel, 2014) and Singularity ( Kurtzer *et al.*, 2017) containerisation solutions.

Table 3 contains a list of relevant software tool versions and sources.

**Table 3.** Software tools: versions and sources.

Software tool	Version	Source
BlobToolKit	4.2.1	https://github.com/blobtoolkit/blobtoolkit
BUSCO	5.3.2	https://gitlab.com/ezlab/busco
CheckM	1.2.1	https://github.com/Ecogenomics/CheckM
DAS Tool	1.1.5	https://github.com/cmks/DAS_Tool
GTDB-TK	2.3.2	https://github.com/Ecogenomics/GTDBTk
Hifiasm	0.16.1-r375	https://github.com/chhylp123/hifiasm
HiGlass	1.11.6	https://github.com/higlass/higlass
Merqury	MerquryFK	https://github.com/thegenemyers/MERQURY.FK
metaBAT	2.15-15-gd6ea400	https://bitbucket.org/berkeleylab/metabat/src/master/
metaMDBG	-	https://github.com/GaetanBenoitDev/metaMDBG
MitoHiFi	2	https://github.com/marcelauliano/MitoHiFi
PretextView	0.2	https://github.com/wtsi-hpag/PretextView
PROKKA	1.14.5	https://github.com/tseemann/prokka
purge_dups	1.2.3	https://github.com/dfguan/purge_dups
YaHS	yahs-1.1.91eebc2	https://github.com/c-zhou/yahs

### Wellcome Sanger Institute – Legal and Governance

The materials that have contributed to this genome note have been supplied by a Tree of Life collaborator. The Wellcome Sanger Institute employs a process whereby due diligence is carried out proportionate to the nature of the materials themselves, and the circumstances under which they have been/are to be collected and provided for use. The purpose of this is to address and mitigate any potential legal and/or ethical implications of receipt and use of the materials as part of the research project, and to ensure that in doing so we align with best practice wherever possible. The overarching areas of consideration are:

•   Ethical review of provenance and sourcing of the material

•   Legality of collection, transfer and use (national and international)

Each transfer of samples is undertaken according to a Research Collaboration Agreement or Material Transfer Agreement entered into by the Tree of Life collaborator, Genome Research Limited (operating as the Wellcome Sanger Institute) and in some circumstances other Tree of Life collaborators.

## Data Availability

European Nucleotide Archive: *Rhynchopus euleeides*. Accession number PRJEB62740; https://identifiers.org/ena.embl/PRJEB62740. The genome sequence is released openly for reuse. The *Rhynchopus euleeides* genome sequencing initiative is part of the Aquatics Symbiosis Genomics (ASG) project ( https://www.ebi.ac.uk/ena/browser/view/PRJEB43743). All raw sequence data and the assembly have been deposited in INSDC databases. Raw data and assembly accession identifiers are reported in Table 1.

## Author information

Members of the Wellcome Sanger Institute Tree of Life Management, Samples and Laboratory Team are listed here: https://doi.org/10.5281/zenodo.10066175.

Members of the Wellcome Sanger Institute Scientific Operations: Sequencing Operations are listed here: https://doi.org/10.5281/zenodo.10043364.

Members of the Wellcome Sanger Institute Tree of Life Core Informatics team are listed here: https://doi.org/10.5281/zenodo.10066637.

Members of the European Bioinformatics Institute ASG Data Portal team are listed here: https://doi.org//10.5281/zenodo.10076466.

Members of the Wellcome Sanger Institute/Aquatic Symbiosis Genomics Project Leadership are listed here: https://doi.org/10.5281/zenodo.10184833.
