# Supplementary material for: The genome sequences of the diplonemid protist Rhynchopus euleeides YPF1915 and its bacterial endosymbiont Candidatus Syngnamydia salmonis (Chlamydiota)
Source: Wellcome Open Res. 2025 May 7;10:233. [Version 1] doi: 10.12688/wellcomeopenres.24014.1 (PMC12171722; doi:10.12688/wellcomeopenres.24014.1)
Supplement: Peer review history

# Reviewer report: Reviewer response for version 1

**DOI:** 10.21956/wellcomeopenres.26494.r123873

**Published:** 2025-06-16

**Authors:** Jack Chi-Ho Ip

**Affiliations:** 1 Science Unit, Lingnan University, Hong Kong, Hong Kong

1. what is the function of endosymbiont *Candidatus Syngnamydia salmonis *in *R. euleeides?*

 2. present the statistical data of mitochondrial scaffolds

 3. BUSCO score is low, compared with other Diplonemidae genome, explain or improve the assembly? or run the eukaryotic BUSCO set?

 4. provide more details about the symbiont genome, which is the key part in title too.

 5. provide the details in lib prep of HiFi and HiC.

 6. show the version of all bioinformatic tools and their settings.

Are sufficient details of methods and materials provided to allow replication by others?

Partly

Is the rationale for creating the dataset(s) clearly described?

Yes

Are the datasets clearly presented in a useable and accessible format?

Yes

Are the protocols appropriate and is the work technically sound?

Yes

Reviewer Expertise:

Molecular ecology and genome analysis

I confirm that I have read this submission and believe that I have an appropriate level of expertise to confirm that it is of an acceptable scientific standard.

# Reviewer report: Reviewer response for version 1

**DOI:** 10.21956/wellcomeopenres.26494.r123870

**Published:** 2025-06-16

**Authors:** Michele Castelli, Luca Erriquez

**Affiliations:** 1 University of Pavia, Pavia, Italy; 2 Department of Biology and Biotechnology, University of Pavia (Ringgold ID: 111209), Pavia, Lombardia, Italy

The work by Tashyreva and co-authors entitled “The genome sequences of the diplonemid protist *Rhynchopus euleeides* YPF1915 and its bacterial endosymbiont *Candidatus* Syngnamydia salmonis (Chlamydiota)” offers a compelling contribution to the field of  genomics of protist and their bacterial symbionts. Thanks to Hi-C, the authors obtained a very high quality (88 chromosomal pseudomolecules) genome assembly of the diplonemid *Rhynchopus euleeides*, a widely distributed yet poorly studied diplonemid. They also assembled its mitogenome and the genome of its chlamydial endosymbiont “ *Candidatus *Syngnamydia salmonis”, though in this case not fully circularized (4 contigs). In any case, given that only one draft diplonemid genome was available until now, this study represents a significant step forward in our understanding of this group. Also the identification of a symbiotic association is noteworthy, offering novel possibilities to improve our understanding of protist-bacterial interactions, especially among such ecologically widespread but neglected host organisms.

 The workflow and data seem very solid, but the writing and presentation could benefit from some improvement, listed below.

1. The structure of the manuscript could be modified to be more clear, e.g. putting the methods immediately after the background, and using heading such as Results, Conclusion (although not strictly required by the Data Note format)
2. The background seems a bit a hybrid between an introduction and a summary. We suggest to put references for data available in the literature, and otherwise include the information in the methods/data results (e.g. species identification of the protist and its endosymbiont, host range for this specific symbiont species)
3. The genome sequence of the symbiont is rightfully emphasised also in the title, but the presentation is relatively concise and should be enriched. The background could explicitly mention any previous knowledge about chlamydial symbioses in diplonemids and other euglenozoans. It could also be reported the precise 16S rRNA gene (mistyped as 18S rRNA gene in the manuscript) identity of the novel isolate with reference sequences of the same attributed species (it is reported only that it was >98.5%, but this is truly a borderline value for bacterial species delimitation, since different authors suggest 98.65-98.7% as threshold). The genome data could be reported more explicitly (size of individual contigs, GC content, BUSCO/CheckM quality metrics). On a side note, these chlamydial contigs seem not immediately visible in the blobtools plot, do they overlap with the protist contigs (based on previous experience, one would expect them to be at relatively lower GC%)?
4. In the methods, an RNA-Seq experiment is mentioned, but it is unclear how this was used in the production of the genomic data obtained. Also, the sequencing methods report both Illumina and PacBio sequencing, while the assembly methods and the results are suggestive of only PacBio used. Please clarify
5. The Hi-C data processing is described in the data analysis method section, but seems to be absent in the description of the laboratory procedures, despite this one being quite detailed. It would be advisable to provide these data for full reproducibility
6. For a full reproducibility, the description of some steps of the assembly procedure could be more refined, for instance reporting full software parameters (e.g. Hifiasm) and mentioning clearly at which step and by which criteria the manual assembly curation, mentioned in the results, was performed
7. The description of the usage of blobtools is very detailed but not very straightforward to be followed, for example it is not entirely clear whether single pseudomolecules were analysed, or if each of them fractionated. As long as this usage is the standard for the software, perhaps the authors could simplify the description of the steps and focus only on the final indices obtained
8. We agree with the authors’ explanation of the low BUSCO scores for the genome, despite its high quality. This conclusion could be fortified by providing, as comparison, scores also for the other diplonemid genome available ( *Diplonema papillatum*) and possibly other related Euglenozoa. In addition, consistent with the authors’ view, the value for Eukaryota odb is understandably much higher, and could thus be used as a more meaningful reference.
9. While it is clear that detailed comparisons between the two haplotype are beyond the scope of a Data note, it would be meaningful to report some more information of the second haplotype, e.g., how were chromosome assigned to each set? is the chromosome size consistent with the first one?
10. The presentation of the mitogenome could be also improved, by summarizing its size and number of molecules in the main text and not only in the table. As a detail, the reference to multipartite nature of this genome (Valach et al. 2017) could be more clearly presented, since in its current version it suggests that the mitogenome of this precise species was analysed therein, rather than, presumably, other diplonemids

Are sufficient details of methods and materials provided to allow replication by others?

Partly

Is the rationale for creating the dataset(s) clearly described?

Yes

Are the datasets clearly presented in a useable and accessible format?

Partly

Are the protocols appropriate and is the work technically sound?

Yes

Reviewer Expertise:

Evolutionary biology of protists and their bacterial symbionts, comparative genomics, phylogeny

We confirm that we have read this submission and believe that we have an appropriate level of expertise to confirm that it is of an acceptable scientific standard.

# Reviewer report: Reviewer response for version 1

**DOI:** 10.21956/wellcomeopenres.26494.r123420

**Published:** 2025-05-30

**Authors:** Alexander K. Tice

**Affiliations:** 1 Mississippi State University, Mississippi, USA

This data note describes the first nuclear and mitochondrial genome sequences of the diplonemid *Rhynchopus euleedides *along with the genome of its symbiont Ca. Syngnamydia salmonis. The methods used to produce and annotate the assembly are modern and of high quality. Researchers in the fields of evolutionary biology, comparative genomics, phylogenomics and more will find these data of great value considering so few genomes are available from this area of the eukaryotic tree of life.

Are sufficient details of methods and materials provided to allow replication by others?

Yes

Is the rationale for creating the dataset(s) clearly described?

Yes

Are the datasets clearly presented in a useable and accessible format?

Yes

Are the protocols appropriate and is the work technically sound?

Yes

Reviewer Expertise:

evolutionary biology, protistology, comparative genomics, phylogenomics, microbial interactions

I confirm that I have read this submission and believe that I have an appropriate level of expertise to confirm that it is of an acceptable scientific standard.
